# TLNPMD: Prediction of miRNA-Disease Associations Based on miRNA-Drug-Disease Three-Layer Heterogeneous Network

**DOI:** 10.3390/molecules27144371

**Published:** 2022-07-07

**Authors:** Yi Yang, Junliang Shang, Yan Sun, Feng Li, Yuanyuan Zhang, Xiang-Zhen Kong, Shengjun Li, Jin-Xing Liu

**Affiliations:** 1 School of Computer Science, Qufu Normal University, Rizhao 276826, China; yangyi5471607@163.com (Y.Y.); sunyan225@126.com (Y.S.); lifeng1028@qfnu.edu.cn (F.L.); kongxzhen@163.com (X.-Z.K.); qfnulsj@163.com (S.L.); sdcavell@126.com (J.-X.L.); 2 School of Information and Control Engineering, Qingdao University of Technology, Qingdao 266520, China; zhangyuanyuan@qut.edu.cn

**Keywords:** miRNA-disease, three-layer heterogeneous, drug heuristic information, network path

## Abstract

Many microRNAs (miRNAs) have been confirmed to be associated with the generation of human diseases. Capturing miRNA–disease associations (M-DAs) provides an effective way to understand the etiology of diseases. Many models for predicting M-DAs have been constructed; nevertheless, there are still several limitations, such as generally considering direct information between miRNAs and diseases, usually ignoring potential knowledge hidden in isolated miRNAs or diseases. To overcome these limitations, in this study a novel method for predicting M-DAs was developed named TLNPMD, highlights of which are the introduction of drug heuristic information and a bipartite network reconstruction strategy. Specifically, three bipartite networks, including drug–miRNA, drug–disease, and miRNA–disease, were reconstructed as weighted ones using such reconstruction strategy. Based on these weighted bipartite networks, as well as three corresponding similarity networks of drugs, miRNAs and diseases, the miRNA–drug-disease three-layer heterogeneous network was constructed. Then, this heterogeneous network was converted into three two-layer heterogeneous networks, for each of which the network path computational model was employed to predict association scores. Finally, both direct and indirect miRNA–disease paths were used to predict M-DAs. Comparative experiments of TLNPMD and other four models were performed and evaluated by five-fold and global leave-one-out cross validations, results of which show that TLNPMD has the highest AUC values among those of compared methods. In addition, case studies of two common diseases were carried out to validate the effectiveness of the TLNPMD. These experiments demonstrate that the TLNPMD may serve as a promising alternative to existing methods for predicting M-DAs.

## 1. Introduction

MicroRNAs (miRNAs) are a class of non-coding RNAs about 22 nucleotides in length, which are involved in a variety of life activities in cells, such as cytogenesis, differentiation, and signal transduction [1,2,3,4]. Up to now, many miRNA-related databases have been compiled and published, which contain a large amount of miRNA-related information. For example, miRbase has collected and recorded the information of about 2500 miRNAs. It has been confirmed that miRNAs have more than 45,000 target gene sites in the early human genome, and more than 60% of the coding genes can be encoded, which can fully demonstrate the extensive regulation of miRNAs [5,6,7]. In recent decades, research reports about the miRNA have been increasing and many miRNA–disease associations (M-DAs) have been confirmed. However, M-DA data is still far from enough, which brings great challenges to traditional biological experiments. Therefore, it is very necessary to put forward scientific and reasonable calculation models. At present, there are many types of methods for M-DA prediction, which can be divided into three categories.

Firstly, based on the assumption that similar miRNAs are more likely to be associated with similar diseases, Xuan et al. constructed a model for predicting M-DAs (MDAPred). The main highlight of MDAPred was to calculate the distance between miRNAs and diseases from the perspective of miRNA clusters and low-dimensional feature space [8]. Shi et al. proposed a framework for predicting M-DAs, which differed from other models by exploiting the relationship between miRNA target proteins and disease genes. The introduction of protein interaction network opened a new path for M-DA prediction [9]. Xuan et al. constructed a model HDMP that integrated the information of semantic and phenotypic disease and miRNA cluster data and comprehensively predicted potential M-DAs by setting more similar miRNAs high weights [10]. To solve the problem that isolated nodes cannot be predicted, Chen et al. proposed a semi-supervised prediction model RWRMDA, which innovatively developed the least squares algorithm to predict all M-DAs without the need for negative samples [11]. Chen et al. proposed a scoring strategy model WBSMDA, which predicted M-DAs by integrating the most similar neighbor nodes of miRNA and disease. The higher the score, the more likely the hidden association between the two [12].

Secondly, based on a complex network, that is, integrating and mapping various heterogeneous data into a network, Chen et al. proposed the HGIMDA model, which calculated the potential M-DAs probability. The final M-DA score was obtained through continuous iterative convergence in the network [13]. Zhao et al. constructed the WINMDA model for predicting hidden M-DAs. WINMDA introduced the shortest path algorithm to construct an interaction network and calculated the M-DA score by calculating the most similar neighbors of miRNA or disease nodes [14]. Chen et al. developed the MNPMDA model, which started from the degree of bias, and used hierarchical clustering and weight transfer strategies to find hidden M-DAs [15].

Thirdly, based on the deep learning framework, Fu et.al. developed a deep ensemble model (DeepMDA). DeepMDA integrated multiple information related to miRNAs and diseases, and firstly constructed features by stacking several deep autoencoders. Finally, DeepMDA used convolutional neural networks to predict M-DA scores [16]. Chen et al. proposed a model, namely DRMDA. DRMDA extracted deep miRNA and disease features based on a variety of information from the two. In addition, after robust feature selection, the final M-DA score was obtained by using support vector machine as a classifier [17]. In order to reduce the influence of only a few related or isolated nodes on the prediction results, Chen et al. proposed a deep belief network model (DBNMDA). Different from other methods, DBNMDA trained the model based on all known M-DAs, and finally obtained the potential M-DA score [18].

In this study, a novel method for predicting M-DAs named TLNPMD was developed, highlights of which are the introduction of drug heuristic information and a bipartite network reconstruction strategy. Specifically, three bipartite networks, including drug–miRNA, drug–disease, and miRNA–disease, were reconstructed as weighted ones using such reconstruction strategy. Based on these weighted bipartite networks, as well as three corresponding similarity networks of drugs, miRNAs and diseases, the miRNA–drug–disease three-layer heterogeneous network was constructed. Then, this heterogeneous network was converted into three two-layer heterogeneous networks, for each of which the network path computational model was employed to predict association scores. Finally, both direct and indirect miRNA–disease paths were used to predict M-DAs. Comparative experiments of TLNPMD and four other models [11,12,13,15,19] were performed and evaluated by five-fold and global leave-one-out cross validations, results of which show that TLNPMD has the highest AUC values among those of compared methods. In addition, case studies of two common diseases were carried out to validate the effectiveness of the TLNPMD. These experiments demonstrate that the TLNPMD may serve as a promising alternative to existing methods for predicting M-DAs. This fully proved that TLNPMD was scientifically effective in the prediction of M-DAs.

## 2. Results

### 2.1. Evaluation Metrics and Performance Comparison

**Figure 1:**
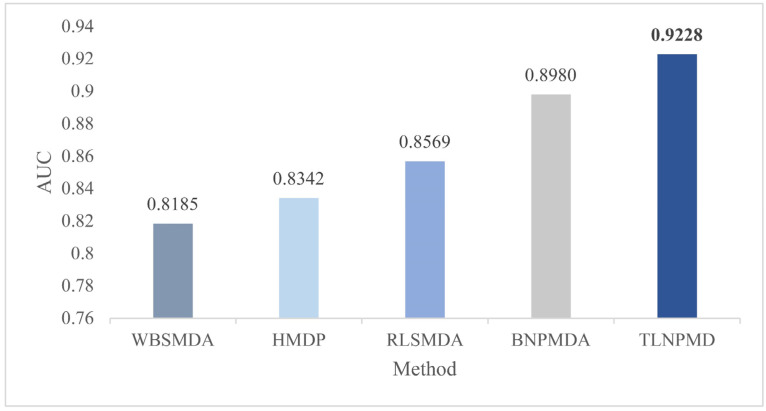
Results of compared models in terms of FCV.

**Figure 2:**
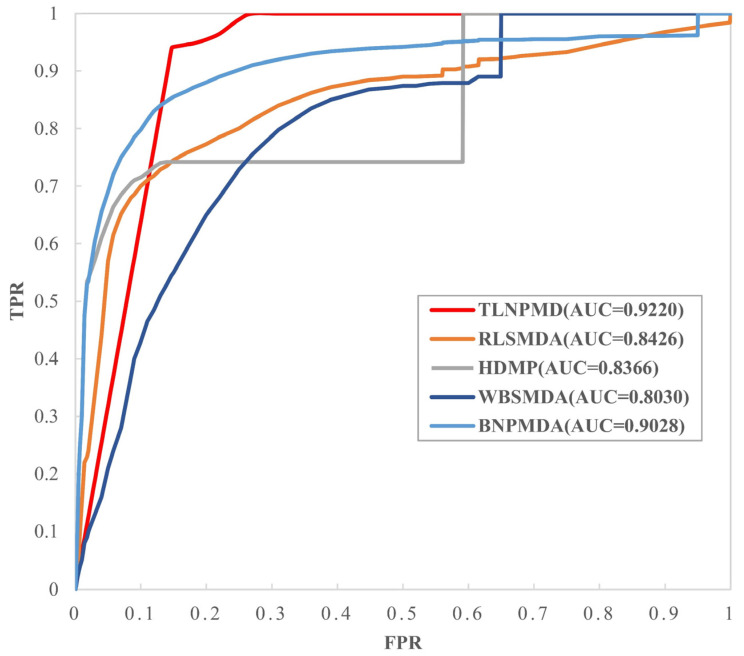
Results of compared models in terms of LOOCV.

We used the fivefold cross-validation (FCV) and global leave out of cross-validation (LOOCV) to verify the capability of TLNPMD. In the FCV, the original M-DAs matrices downloaded from the HMDDv2.0 database were randomly divided into five groups, then all M-DAs in one group were set to 0 as the test sample set, and the other four groups remained unchanged as the training sample sets. Each group of original matrices was used as a test set once and scored through the TLNPMD. Finally, all M-DA scores could be obtained and then ranked. FCV was performed 100 times and AUC was averaged as the final prediction result of TLNPMD. In global LOOCV, the each test sample was the known M-DA, and the training samples were the remaining M-DAs. TLNPMD was compared with the other four different methods, namely WBSMDA, HDMP, RLSMDA, and BNPMDA through FCV and global LOOCV. These four methods are representative to a certain extent: WBSMDA and HDMP are often used as the basic methods in this research field RLSMDA is a method that applied machine learning to this research field, and BNPMDA is the method with recently published high-level papers. The AUC of the FCV were calculated for all the methods using the same data. The comparison results are shown in Figure 1. The AUC value of WBSMDA was 0.8185, HMDP was 0.8342, RLSMDA was 0.8569, BNPMDA was 0.8980. TLNPMD was 0.9228, which was significantly higher than other four methods. Furthermore, we also compared TLNPMD with another four methods for the global LOOCV; including TLNPMD, BNPMD, WBSMDA, HDMP, and RLSMDA AUC values were 0.9220, 0.9028, 0.8030, 0.8366 and 0.8426, respectively. Clearly, TLNPMD performed best, and the above results are shown in Figure 2.

### 2.2. Effects of Parameters

**Figure 3:**
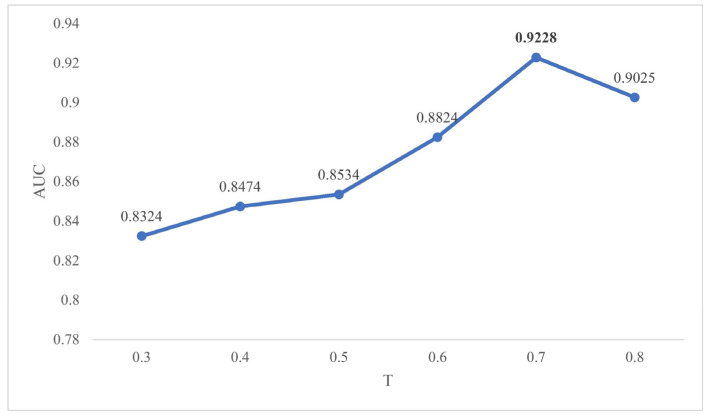
The AUC values of FCV with T being 0.3–0.8.

**Figure 4:**
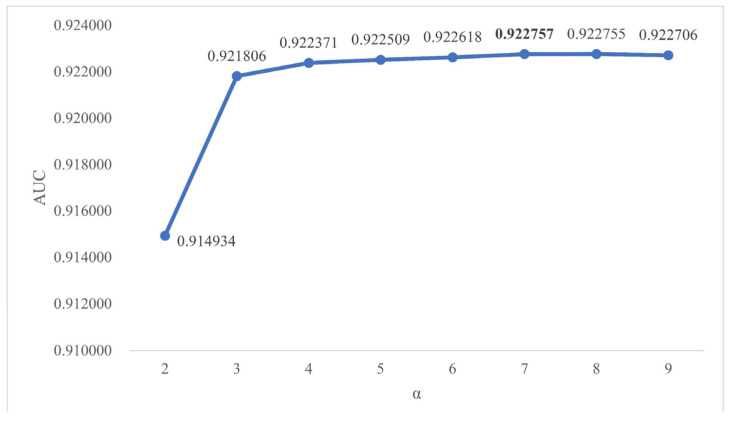
The AUC results of FCV when α with different values.

In the method section, we mentioned three parameters, which were the path length L, threshold parameter T and attenuation factor α. Considering that the longer the path is, the greater the chance that the error may be generated, we set the path length as 2. In this way, TLNPMD not only avoided the closed-loop path, but also greatly reduced the time complexity of the algorithm. The threshold parameter T and attenuation α were finally determined through continuous experimental adjustment. In the range of 0.3 to 0.8, we adjusted the value of T according to the rule that the step size was 0.1. The ROC curve under FCV was shown in Figure 3 when T was set to different values. We adjusted the value of α according to the rule that the step size was 1.0 in the range of 2.0 to 7.0; the AUC values under FCV are shown in Figure 4. Through the experimental results, we finally determined to set T as 0.7 and α as 7.0.

### 2.3. Case Study

After we obtained the final M–DAs score through the TLNPMD, we selected hepatocellular carcinoma (HCC) and breast neoplasms (BN) from 383 diseases for case study. Based on the final predicted result, we ranked the scores of 495 miRNAs for the two diseases and selected the top 20 miRNAs, respectively. We searched the associations between these miRNAs and two different diseases one by one to confirm whether there were associations in the HMDD v3.2 and miRcancer; the records are shown in Table 1 and Table 2, respectively. The reasons for choosing these two diseases are as follows.

HCC is a common tumor which has multiple etiologies, such as viral hepatitis infection and fatty liver [20]. Liver cancer is one of the five most common cancers in the world and has caused great trouble to human health, with a high mortality rate, most of which are HCC related [21]. In recent years, a growing number of miRNAs associated with HCC have been successively confirmed. Shen et al. experimentally proved that miRNA-10a-5p acted on SKA1 to inhibit the metastasis of hepatocellular carcinoma cells [22]. Liu et al. demonstrated that miRNA-494 can promote cell proliferation in HCC [23]. Bandiera S et.al confirmed that miRNA-122 was inextricably linked with liver cancer and was an important cause of liver cancer [24].

BN is one of the five most common tumors in the world, and its main diagnosis population is women, which is extremely harmful. According to statistics, the incidence of BN has increasing by 3.1% every year, which is a serious threat to human health [25]. Nowadays, the diagnosis of BN is mainly through ultrasound, a scientific and effective method to study its pathogenesis. MiRNAs play an important role in the regulation of cellular genes and exploring the relationship between miRNA and BN is of great significance for preventing and treating BN [26]. In recent years, many miRNAs have been confirmed to be in association with BN. Mansoori et al. demonstrated that the miR-142-3p directly targets the 3′ untranslated region of HMGA2, which encodes an onco-embryonic protein that is overexpressed in most cancers, including BN [27]. Silvia et al. demonstrated that vav1 promoted transcription of mature miR-29b in breast cancer cells. [28].

According to the above description, these two diseases are very common. It is very important to find the treatment for these two diseases. Table 1 and Table 2 show most of the top 20 miRNAs in association with these two diseases, obtained by TLNPMD. This also fully explains the performance of TLNPMD.

## 3. Discussion

In this study, a novel method for predicting M-DAs named TLNPMD was developed, highlights of which are the introduction of drug heuristic information and a bipartite network reconstruction strategy. Specifically, three bipartite networks, including drug–miRNA, drug–disease, and miRNA–disease were reconstructed as weighted ones using such reconstruction strategy. Based on these weighted bipartite networks, as well as three corresponding similarity networks of drugs, miRNAs and diseases, the miRNA-drug-disease three-layer heterogeneous network was constructed. Then, this heterogeneous network was converted into three two-layer heterogeneous networks, for each of which the network path computational model was employed to predict association scores. Finally, both direct and indirect miRNA-disease paths were used to predict M-DAs.

The advantages of TLNPMD can be summarized as follows: (1) the data sets used for prediction are scientific and reliable; (2) the methods of data integration and alignment were reasonable; (3) the bipartite network reconstruction strategy increased network complexity; (4) a three-layer heterogeneous network was constructed, and drug nodes were added, which meant that a reliable path was added when the M-DAs were predicted through network path. 

Of course, TLNPMD still has some shortcomings. Firstly, although it reduced the dependence on the known M-DAs, it has not completely eliminated this dependency. Secondly, all data had sparsity problems, which greatly affected the performance of TLNPMD. Finally, in the determination of parameter threshold, deep learning methods could be considered for improvement.

## 4. Materials and Methods

### 4.1. The Space of MiRNA

By assuming that miRNAs with more similar functions are associated with more similar diseases, the MISIM database [29] was compiled, which contains all the miRNA-related information required by TLNPMD. We downloaded the miRNA functional similarity information from MISIM and organized it into a matrix MRF, where MRF(m,n) denoted the similarity of between miRNA m and miRNA n.

### 4.2. The Space of Drug

Based on the theory that, the more similar different drugs are, the more similar their chemical structure, the Chemical Development Kit [30] calculates the similarity score between different drugs. We downloaded the data and transformed it into matrix DCS, which contains the relationship of 662 drugs. DCS(i,j) represents the similarity of drug i to drug j.

### 4.3. The Space of Disease

#### 4.3.1. Model 1

The MeSH database is a disease classification system that provides information on different diseases. Relationship between different diseases is measured by constructing a directed acyclic graph (DAG), which can be obtained by MeSH [31]. The DAG of disease L was expressed as $DAG_{L}=\left\{N_{L},E_{L}\right\}$, where $N_{L}$ denoted disease L and all its ancestor nodes and $E_{L}$ represented all connected edges between different diseases in the $DAG_{L}$. We calculated the semantic contribution of the disease semantic block a in $DAG_{L}$ and the calculation method is as follows [32]:

(1) $$MOD1\left(a\right)=\{\begin{array}{c} 1\begin{array}{cc} & a=L \end{array}\\ \max\left(\Phi \times MOD1_{L}\left(a\prime \right)|a\prime \in C\left(a\right)\right)\begin{array}{cc} & a\neq L \end{array} \end{array}$$

where C(a) is all child nodes of semantic block a, Φ denotes the semantic contribution factor of the edges connecting N to N′ in $E_{L}$ and usually is set to 0.5 [29]. The final semantic value of L is calculated as:

(2) $$MOD1\left(L\right)=\sum_{a\in N_{L}}MOD1_{L}\left(a\right)$$

The similarity of two diseases is calculated by Model 1, the calculation is as follows:

(3) $$DDS1\left(L,M\right)=\frac{\sum_{a\in N_{L}\cap N_{M}}MOD1_{L}\left(a\right)+MOD1_{M}\left(a\right)}{MOD1\left(L\right)+MOD1\left(M\right)}$$

where DDS1 denoted the similarity information of different diseases calculated by Model 1.

#### 4.3.2. Model 2

In Model 1, there is a disadvantage that the disease blocks in the same layer of a specific disease have the same contribution value to this disease. To improve this problem, another model is proposed [10], which defines the semantic contribution value of a in $DAG_{L}$ as follows:

(4) MOD2(a)=−log(DAG(a)/N)

where DAG(a) is used to represent the number of all diseases with a present in the DAGs. The final semantic value of L is calculated as:

(5) $$MOD2\left(L\right)=\sum_{a\in N_{L}}MOD2_{L}\left(a\right)$$

If the similarity of two diseases is calculated by Model 2, the detail is as follows:

(6) $$DDS2\left(L,M\right)=\frac{\sum_{a\in N_{L}\cap N_{M}}MOD2_{L}\left(a\right)+MOD2_{M}\left(a\right)}{MOD2\left(L\right)+MOD2\left(M\right)}$$

In TLNPMD, to make the calculation results more comprehensive, Model 1 and Model 2 are combined and averaged as the final similarity representation between different diseases:

(7) $$FDS\left(L,M\right)=\frac{DDS2\left(L,M\right)+DDS2\left(L,M\right)}{2}$$

### 4.4. The Space of Interaction

In the interaction space, the main data sets of known association are included, namely the known M-DAs, miRNA–drug associations (MIDAs), and drug–disease interactions (DDIs). 

The information of known M-DAs was downloaded from HMDD v2.0, which contained 495 miRNAs, 383 diseases, and 5430 experimentally confirmed associations between them.

Following the above, we have determined 495 miRNAs, 383 diseases, and 662 drugs. In order to obtain the relationship between miRNAs and drugs, we searched the SM2miR database (http://www.jianglab.cn/SM2miR/: accessed on 22 March 2021) and the Pharmacomi (https://originalsteroids.org/brand/pharmacom: accessed on 22 March 2021) database, which contains considerable MIDAs information, and finally obtained 992 confirmed MIDAs.

For all DDIs information, we comprehensively queried not only the gold standard dataset Fdataset [33], but also the Cdataset database [34], and finally obtained 799 known interactions between 662 drugs and 383 diseases.

### 4.5. Network Reconstruction

After the above steps, all data, including three similarity relationship (namely miRNA–miRNA, drug–drug and disease–disease), three association relationship (namely M-DAs, MIDAs and DDIs) were processed, and S1, S2, S3, A1, A2 and A3 were used to represent them, respectively.

In most of the current methods for predicting potential M-DAs based on the network, many isolated nodes—that is, nodes without any associations—were not fully utilized. In order to solve this problem, inspired by Chen et al. [35], we introduced a network reconstruction strategy. Three association relationships were mapped to three bipartite networks, namely A1, A2 and A3, and they were reconstructed by this strategy. Taking the specific analysis of the A1 as an example, the strategy for reconstructing is as follows:

(8) $$A1\prime \left(a,c\right)=\sum_{i=1}^{p}\sum_{j=1}^{r}S1\left(a,a_{i}\right)\times S3\left(c,c_{j}\right)\times A1\left(a_{i},c_{j}\right)$$

where a and c denote a specific miRNA and a specific disease, respectively, the number of them are represented as p and r. Considering the weak similarity nodes of a or c may influence the accuracy of results, the parameter T was set. The nodes with a similarity less than T to a or c were removed. However, as all the neighbor nodes of some special miRNAs or diseases may be deleted because the similarity of all was less than T, we retained the most similar node with this kind of miRNA or disease. 

The same reconstruction strategy for A2 is as follows:

(9) $$A2\prime \left(a,b\right)=\sum_{i=1}^{p}\sum_{j=1}^{q}S1\left(a,a_{i}\right)\times S2\left(b,b_{j}\right)\times A2\left(a_{i},b_{j}\right)$$

where b is a specific drug and q represents the number of drugs. The strategy for reconstructing A3 is as follows:

(10) $$A3\prime \left(b,c\right)=\sum_{i=1}^{q}\sum_{j=1}^{r}S2\left(b,b_{i}\right)\times S3\left(c,c_{j}\right)\times A3\left(a_{i},b_{j}\right)$$

### 4.6. Three-Layer Heterogeneous Network Construction

Reconstructed three bipartite networks edge weights were random, so the normalization processing was necessary. Taking the specific analysis of the A1 as an example, the method is as follows:

(11) $$A1^{\prime \prime }=A1^{\prime }/\max\left(A1^{\prime }\right)$$

We picked the largest reconstruction weight in the $A1^{\prime \prime }$, and then used all reconstruction weights to carry out a quotient with it. $A2^{\prime \prime }$ and $A3^{\prime \prime }$ were obtained by the same calculation process. Finally, three reconstructed bipartite networks and three similarity networks—namely $A1^{\prime \prime }$, $A2^{\prime \prime }$, $A3^{\prime \prime }$, S1, S2, and S3—were fused into the three-layer heterogeneous network, which was denoted $TN^{\prime }$.

(12) $$TN^{\prime }=\left[\begin{array}{c} S1\\ A1^{\prime \prime }\\ S2 \end{array}\begin{array}{c} A2^{\prime \prime }\\ S3\\ A3^{\prime \prime } \end{array}\right]$$

### 4.7. Prediction of M-DAs

**Figure 5:**
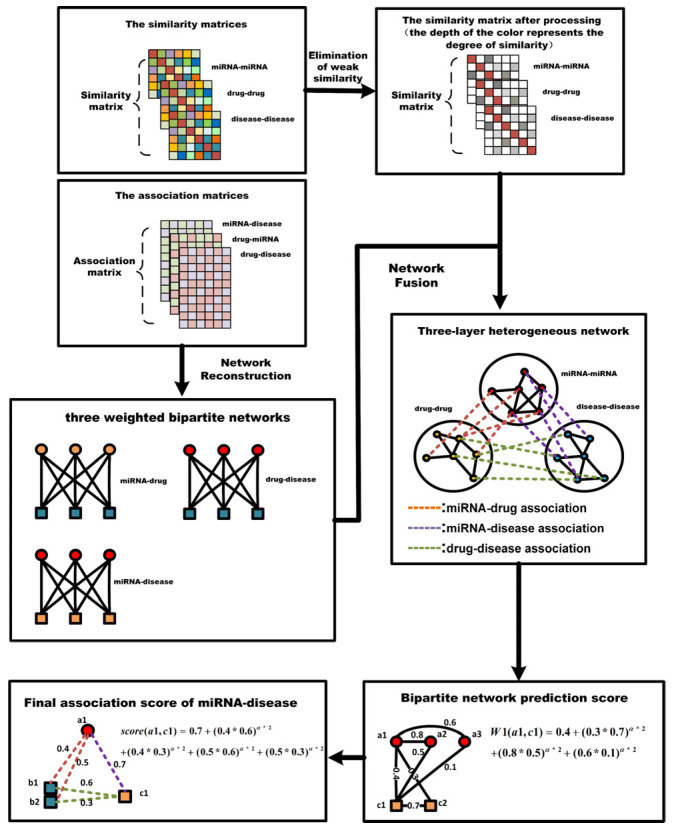
Flowchart of TLNPMD.

In this study, we aimed to predict the potential of M-DAs based on the three-layer heterogeneous network path. For a clear description, we divided TLNPMD into two parts: (i) the two-layer network internal path search and (ii) the overall path search of the three-layer network. The flowchart of TLNPMD was shown in Figure 5.

For two-layer network internal path search, $TN^{\prime }$ was seen as three two-layer network, namely miRNA–disease, miRNA–drug, and drug–disease. Inspired by PBMDA [36], a path-based model was improved and the two-layer network of miRNA–disease used as an example to describe it in detail. The similarity part adopted the above method to delete the network of weak similarity nodes. The depth-first search algorithm was introduced to search the path between miRNA nodes and disease nodes. The path length parameter L was set to 2, which had three reasons: (i) to reduce the time complexity of the algorithm; (ii) to avoid the formation of a closed-loop search path; (iii) to obtain accurate results after many times experiment. The final path weight between a miRNA node and a disease node represented the possibility of association and the calculation formula was defined as follows:

(13) $$W1\left(a,c\right)=A1^{\prime \prime }\left(a,c\right)+{\sum_{w=1}^{n}\left(\prod p_{w}\right)}^{fdecay\left(p_{w}\right)}$$

where $\prod p_{w}$ is the product of two path weights, which described the path of length 2. If the path from a specific miRNA node to a specific disease node was longer, it is more likely to produce errors, so we introduced a decay function fdecay, which is defined as follows:

(14) fdecay(p)=β×len(p)

In TLNPMD, len(p) is limited to 2 and β is set to 7.0 after many experiments. After the above steps, the path weight of each miRNA-disease pair was obtained. Following the same steps, other two networks were calculated.

(15) $$W2\left(a,b\right)=A2^{\prime \prime }\left(a,b\right)+{\sum_{w=1}^{n}\left(\prod p_{w}\right)}^{fdecay\left(p_{w}\right)}$$

(16) $$W3\left(b,c\right)=A3^{\prime \prime }\left(b,c\right)+{\sum_{w=1}^{n}\left(\prod p_{w}\right)}^{fdecay\left(p_{w}\right)}$$

For the overall path search of the three-layer network, two paths from miRNA nodes to disease nodes were searched in three-layer heterogeneous $TN^{\prime }$, namely the direct connection path and the indirect path base on drug nodes as the medium. These two paths were integrated to obtain the final weight, which was the score of M-DAs. The calculation formula is follows:

(17) $$Score\left(a,c\right)=W1\left(a,c\right)+{\left(W2\left(a,b\right)\times W3\left(b,c\right)\right)}^{fdecay\left(p\right)}$$

## 5. Conclusions

With the advancement and improvement of technology, many miRNAs have been confirmed to be associated with the generation of human various diseases. Capturing M-DAs provides an effective way to gain insight into the etiology of human diseases and accurately treat them. Many models for predicting M-DAs have been constructed; nevertheless, there are still several limitations, such as generally considering direct information between miRNAs and diseases and usually ignoring potential knowledge hidden in isolated miRNAs or diseases. To overcome these limitations, in this study a novel method for predicting M–DAs was developed, named TLNPMD, highlights of which are the introduction of drug heuristic information and a bipartite network reconstruction strategy. Specifically, three bipartite networks—including drug–miRNA, drug–disease, and miRNA–disease—were reconstructed as weighted ones using such reconstruction strategy. Based on these weighted bipartite networks, as well as three corresponding similarity networks of drugs, miRNAs and diseases, the miRNA–drug–disease three-layer heterogeneous network was constructed. This heterogeneous network was converted into three two-layer heterogeneous networks, for each of which the network path computational model was employed to predict association scores. Finally, both direct and indirect miRNA–disease paths were used to predict M-DAs. Comparative experiments of TLNPMD and four other models were performed and evaluated by five-fold and global leave-one-out cross-validations, results of which show that TLNPMD has the highest AUC values among the compared methods. In addition, case studies of two common diseases were carried out to validate the effectiveness of the TLNPMD. These experiments demonstrate that the TLNPMD may serve as a promising alternative to existing methods for predicting M-DAs.

## Figures and Tables

**Table 1:** TLNPMD was applied to HCC to predict the top 20 disease-related miRNAs.

miRNA	Evidence
‘mir-515’	HMDD v3.2
‘mir-520a’	HMDD v3.2; miRcancer
‘mir-520h’	HMDD v3.2
‘mir-526a’	HMDD v3.2
‘mir-330′	HMDD v3.2
‘mir-512′	HMDD v3.2
‘mir-520e’	HMDD v3.2
‘mir-526b’	HMDD v3.2; miRcancer
‘mir-297’	HMDD v3.2
‘mir-325’	HMDD v3.2; miRcancer
‘mir-520f’	miRcancer
‘mir-520g’	HMDD v3.2
‘mir-136’	HMDD v3.2
‘mir-300’	HMDD v3.2
‘mir-507’	unconfirm
‘mir-523’	unconfirm
‘mir-525′	HMDD v3.2
‘mir-331’	HMDD v3.2; miRcancer
‘mir-658’	unconfirm
‘mir-134’	HMDD v3.2; miRcancer

**Table 2:** TLNPMD was applied to BN to predict the top 20 disease-related miRNAs.

miRNA	Evidence
‘mir-519b’	miRcancer
‘mir-922’	HMDD v3.2
‘mir-92’	HMDD v3.2; miRcancer
‘mir-1254’	HMDD v3.2
‘mir-630’	HMDD v3.2; miRcancer
‘mir-624’	unconfirm
‘mir-369’	unconfirm
‘mir-661’	HMDD v3.2
‘mir-329’	miRcancer
‘mir-134’	HMDD v3.2; miRcancer
‘mir-574’	HMDD v3.2; miRcancer
‘mir-124a’	HMDD v3.2; miRcancer
‘mir-516a’	HMDD v3.2
‘mir-516b’	HMDD v3.2
‘mir-197’	HMDD v3.2
‘mir-324’	HMDD v3.2
‘mir-629’	HMDD v3.2
‘mir-337’	unconfirm
‘mir-662’	unconfirm
‘mir-486’	HMDD v3.2

## Data Availability

Not applicable.
